# Anthocyanins and Vascular Health: A Matter of Metabolites

**DOI:** 10.3390/foods12091796

**Published:** 2023-04-26

**Authors:** Joseph Festa, Aamir Hussain, Zakia Al-Hareth, Harprit Singh, Mariasole Da Boit

**Affiliations:** 1 Leicester School of Allied Health Sciences, De Montfort University, The Gateway, Leicester LE1 9BH, UKzakia.alhareth@ndm.ox.ac.uk (Z.A.-H.);; 2 Pandemic Sciences Institute, Old Road Campus, University of Oxford, Roosevelt Drive, Headington, Oxford OX3 7TY, UK

**Keywords:** anthocyanins, metabolites, oxidative stress, inflammation, endothelial dysfunction

## Abstract

Anthocyanins are a subgroup of flavonoid polyphenols previously investigated for improving cardiovascular health and preventing the development of endothelial dysfunction. However, their poor bioavailability raises the question of whether the observed biological activity is due to their metabolites. Phenolic metabolites can reach higher plasma concentrations and can persist in the circulation for periods much longer than their original anthocyanin form; therefore, the biological activity and health promoting effects of anthocyanins may differ from their metabolites. To address this, recent studies have facilitated different cell models, in vivo studies and explored physiologically relevant concentrations to better understand their mechanisms of action. The criteria were chosen based on previous reports demonstrating that anthocyanins can improve endothelial function via modulation of the Akt-endothelial nitric oxide synthase pathway and transcription factors Nrf2 and NF-κB, which made it critical to assess the phenolic metabolites’ modes of action via these pathways. This review demonstrates how phenolic metabolites differ in bioactivity from their precursor anthocyanin, demonstrating improved endothelial function in response to inflammatory mediators at concentrations that are tolerated in vivo. The review highlights the crucial need for further studies to focus on improving the bioavailability of metabolites in isolation and explore the effect of metabolites in mixtures.

## 1. Introduction

The increased occurrence of cardiovascular disease (CVD) over the last 25 years, with one in four deaths in Europe, has presented CVD as a public health priority, particularly in the prevention through lifestyle interventions. Although daily recommendations for healthier lifestyle choices are of high importance for patients with CVD comorbidities, there are many implications and aspects to consider. Endothelial dysfunction (ED) is an independent prognosticator of cardiovascular events playing a vital role in the initiation of atherosclerosis and the progression of clinical complications consistently associated with diabetes, obesity, and hypertension [1].

**Figure 1:**
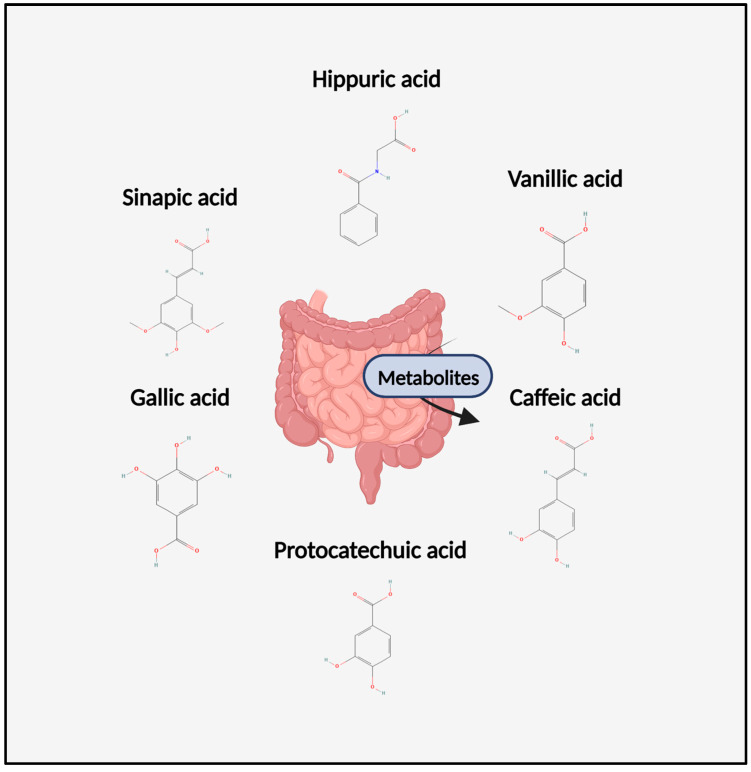
A diagram of the chemical structures of metabolites discussed throughout this review. Figure was created with Biorender.com.

Recently, the consumption of anthocyanins, which are a subgroup of flavonoid polyphenols, has been associated with reduced CVD mortality [2]. More than two-thirds of trials have reported beneficial effects of anthocyanins derived from fruits on the markers of CVD risk, including endothelial function [3,4]. Despite their overall health promoting effects, anthocyanins have very low bioavailability, less than 1% detected in vivo, which is translated to plasma concentrations of less than 100 nM [5,6]. This could be related to the instability of anthocyanins at physiological pH, which are usually present in plasma for 1–4 h before degradation [5,6]. On the contrary, the degradation products of anthocyanins, known as phenolic metabolites, can be found in the circulation at concentrations higher than their original forms (<42-fold) and can be detected up to 48 h after consumption of many fruits and vegetables [7,8]. In healthy men, the consumption of 240 g of blueberries increased flow-mediated dilation (FMD) at 1–2 and 6 h post-consumption [8]. This was strongly linked to the increase in phenolic metabolites found in plasma, including ferulic acid, isoferulic acid, vanillic acid, 2-hydroxybenzoic acid, benzoic acid, and caffeic acid (sum of conjugated and non-conjugated compounds), that reached a total plasma concentration of 0.4 µM [8]. Studies now reveal that phenolic metabolites’ presence in plasma correlates with improved markers of endothelial function, suggesting that the metabolites are at the forefront of the observed biological activity. Considering these findings, studies are currently establishing better identification of the benefits of anthocyanins, through investigating the mechanism of action of phenolic metabolites, leading to a clearer understanding of application in vivo. Thereby, the focus of the present review is to highlight the recent research on specific anthocyanins’ phenolic metabolites for improving vascular function (Figure 1), in addition to showing how the biological activity may differ between the precursor and metabolite products at physiologically relevant concentrations.

## 2. Method and Literature Search

A literature search was completed on PubMed, Scopus, and Google websites which included all original research articles written in English on “anthocyanin metabolites and endothelial function/vascular health”. Keywords included anthocyanin metabolites, endothelial function, vascular health, and inflammation. Human trials were only selected based on data exhibiting phenolic metabolites of anthocyanins, studies only reporting anthocyanins were not included throughout the current review.

## 3. Metabolism of Anthocyanins

Anthocyanins are water-soluble, glycosylated, and non-acetylated polyphenolic compounds that form the red, blue, and purple pigments of fruits [9]. There are over 700 different types of anthocyanin found in nature but only six anthocyanidins: cyanidin, delphinidin, pelargonidin, peonidin, malvidin, and petunidin, are widely distributed in the human diet [10]. The anthocyanidin types differ in the number of hydroxyl groups attached to their ring structure, degree of methylation, type, and the number of sugar molecules (mono-, di-, or tri-glycosides), and number of aliphatic or aromatic acids, which enable them to scavenge reactive oxygen species (ROS) directly [11]. The structure of anthocyanins plays a critical role in determining the extent of degradation caused by saliva in the mouth. This is largely governed by oral microbiota glycosides in the form of mono, di, and tri that are susceptible to this first-line degradation phase [12]. After ingestion, anthocyanins are absorbed by the stomach lining and are rapidly present in the bloodstream, reaching maximum concentrations of around 0.1 μM within the first 1–3 h [13]. At 4 h following consumption, 60–90% of the original anthocyanins may disappear from the gastrointestinal tract where they are transformed into metabolites [14,15]. The gastrointestinal conditions are influential in the stability of anthocyanin degradation products, as pH and temperature can cause significant degradation depending on the structure of the anthocyanin, such as the methoxy group on the B ring has been shown to improve stability whereas the hydroxyl group and acylation reduce stability [16]. When anthocyanins reach the small intestine, they degrade to glucuronic, methylated, and sulfate metabolites in the liver which are known as phase-2 metabolites and peak in plasma (<1 μM) at 3–5 h [7,17]. The above degradation products are catalyzed by the following enzymes: uridine diphosphate-glucuronosyltransferases, sulfotransferases, and catechol-O-methyltransferases [18]. Nevertheless, it is still possible for a low percentage of anthocyanins such as cyanidin-3-glucoside (C3G) and pelargonidin-3-glucoside to be absorbed into the gastrointestinal wall in their original unmetabolized form [16].

Unabsorbed anthocyanins are then extensively metabolized by the intestinal microbiota in the colon, giving rise to phenolic acids, including protocatechuic acid (PCA) and phloroglucinaldehyde, which are derived from the A and B rings of the parental compound [19]. Phenolic acids can also go through methylation, which alters the number of hydroxyl and methoxyl groups in ring B compared to the precursor compound [17]. Phenolic acids that have been methylated, such as vanillic acid (VA) or ferulic acid, can reach peak plasma concentration (1–2 µM) within 15 h, in addition to being detected in plasma up to 48 h after ingestion [7]. Following berry ingestion, metabolites can reach substantial concentrations in some participants (1–40 µM) with a diverse mixture of glucuronide and sulfate isomers ranging between 0.01 and 0.35 µM [20]. Moreover, the gut microbiota plays an important role in the metabolism and bioavailability of phenolic metabolites. When not absorbed by the small intestine they can reach the large intestine through being subjected to fermentation by gut microbiota. The fermentation can result in various metabolites: short chains fatty acid, phenolic acid, and urolithins. The gut microbiota can also affect the bioavailability of anthocyanins by modifying their chemical structure through enzymatic transformations. For example, some bacteria in the gut can convert flavonoids into more bioavailable forms, such as aglycones, which are more easily absorbed in the gut and transported to the bloodstream. Aglycons can enter epithelial cells by passive diffusion, or a sodium-dependent glucose transporter can be involved in the transport of the glycosides.

Due to these differences in bioavailability, some previous in vitro data may be miss-leading because of the prolonged incubations, supra-physiological concentrations, and incubations with anthocyanins or whole extracts. These experimental conditions do not reflect in vivo environments, making it difficult to appreciate the potential health protective properties of anthocyanins [21]. However, more recent studies have instead used concentrations achievable in vivo which allow further understanding of the biological activities of anthocyanins and their phenolic metabolites in relation to vascular health.

## 4. Anthocyanin Metabolites and Endothelial Function In Vivo

In recent years, there has been a growing interest in establishing a correlation of metabolite detection in plasma after the consumption of anthocyanins with improved endothelial function/CVD markers. A recent observational study found that up to 80% of the total dietary anthocyanins are derived from the consumption of berries, wines, and non-alcoholic drinks [22]. In a randomized control clinical trial, adults with moderate hypercholesterolemia who consumed an anthocyanin-rich strawberry drink daily for 4 weeks were associated with an increased FMD at week 0, 1 h post strawberry drink consumption [23]. The improved FMD was linked to an increase in plasma metabolites and, more specifically, selected metabolites were observed to be associated with pre-occlusion diameters which could partly explain the FMD responses [23]. Two additional studies have reported similar findings, demonstrating an increase in FMD responses after the consumption of berry extracts, which were associated with elevated plasma levels of metabolites [24,25]. It is important to note that none of these studies measured nitric oxide (NO) production, despite observing an increase in FMD and plasma levels of metabolites following the consumption of berry extracts. Nitric oxide is a potent vasodilator that plays a crucial role in regulating blood flow and blood pressure, and therefore, its absence in these studies leaves the open question of whether the observed improvements in FMD were directly linked to NO production or due to other mechanisms. However, in a 6-month study, after the consumption of blueberry powder daily, an increase of 1.45% in FMD was seen in middle-aged/older men and women with metabolic syndrome and increases in cyclic guanosine monophosphate [26]. Moreover, after the daily consumption of blueberry powder for 12 weeks improved endothelial function and reduced oxidative stress in postmenopausal women aged 45–65 years with elevated blood pressure, was directly linked to the increase in polyphenol metabolites at 4, 8, and 12 weeks compared to the placebo group [27]. Despite having no change in phosphorylated endothelial nitric oxide synthase (eNOS) expression over the 12 weeks, the blueberry group FMD was inversely associated with NADPH oxidase protein expression and positively associated with phosphorylated eNOS expression [27].

There have been studies in which the plasma metabolites were not associated with improved vascular responses following the intake of energy-dense high-fat/high-sugar meals [28]. However, within this study, the test meal included 65.1 g of fat (including 25.8 g of saturated fat). It has been previously observed that meal consumption per se reduces FMD responses and that postprandial vascular function is significantly impaired following previous high fat intake [28]. Moreover, in a study that included 102 prehypertensive participants, no change in endothelial function was seen following the daily consumption of encapsulated Aronia berry extract [29]. Despite no change in endothelial function following the intervention, improvements in augmentation index (Aix) and pulse wave velocity (PWV) were found in the Aronia group vs the control group. A total of 23 urinary and 43 plasma phenolic metabolites, mainly cinnamic and benzoic acid derivatives, benzene diols, and triols, were significantly correlated with the decreases in PWV and Aix found in the study [29].

A recent systematic and meta-analysis review has demonstrated that Hibiscus sabdariffa can improve CVD markers, including improved blood pressure [30]. This has been directly linked to increases in NO production via eNOS as well as a reduction in proinflammatory markers and oxidative stress [31]. It is now considered likely that the phenolic metabolites derived from hibiscus play a predominant role. Metabolites such as hippuric and gallic acid (GA) have been detected in plasma following the acute intake of hibiscus extract and could explain the previous findings [32]. 

It is possible that the mechanisms underlying the changes in vascular function are related to alterations at the epigenetics level over time. However, it is also likely that the initial changes are related to the modulation of signaling pathways directly linked to FMD responses during the close temporal position. Nevertheless, it should be noted that this finding is not conclusive, and further research is necessary to determine the exact mechanisms underlying these changes. It is important to note that the bioavailability of phenolic metabolites can be influenced by many factors, including their chemical structure, solubility, and interaction with other food components. Therefore, understanding the mechanisms in mouse models or in vitro could help with further clarification for application in vivo.

## 5. Anthocyanin Metabolites, Nitric Oxide Production via Akt-eNOS Pathway

The endothelium is a single layer of cells adjacent to the lumen of blood vessels and is well-defined as a metabolically active organ that has a key role in the control of vascular homeostasis [33,34]. It lines several different types of vessels, each with distinct functions, and the functions of the endothelium vary according to which type of vessel it is lining [33]. It is a barrier between blood and tissue that controls the exchange of nutrients, solutes, hormones, macromolecules, and leukocytes between blood and interstitial compartments. More specifically, endothelial cells (EC) maintain and regulate smooth muscle cell growth, proinflammatory molecules, angiogenesis, modulation of platelet aggregation and coagulation, and vascular tone [34,35].

Damage to the endothelium, also known as ED, disrupts the balance between the release of vasodilators/constrictors and initiates several events that promote atherosclerosis, such as increased endothelial permeability, platelet aggregation, leukocyte adhesion, and the generation of proinflammatory cytokines [34]. ED is the first subclinical sign of atherosclerosis and is associated with many CVD comorbidities [36]. Whilst the mechanisms for ED are multifactorial, the regulation of the endothelium is mainly reliant on the availability of NO [37,38]. Nitric oxide is the primary vasodilator found within the vasculature and plays a central role in the regulation of vascular homeostasis. NO has an unpaired electron, making it highly reactive and, in an environment with the overproduction of ROS, it can initiate the conversion of NO to peroxynitrite (NO + O_2_ = ONOO-), a cytotoxic agent, further contributing to the progression of ED [37,38,39]. Under all conditions in which an absolute or relative NO deficit is encountered, the process of atherosclerosis is initiated or accelerated [35,39]. This suggests that NO bioavailability is important and can be improved by inducing or scavenging the excess production of ROS molecules [40,41]. In ECs, NO is synthesized primarily from the increased expression and phosphorylation of the eNOS enzyme [42].

**Figure 2:**
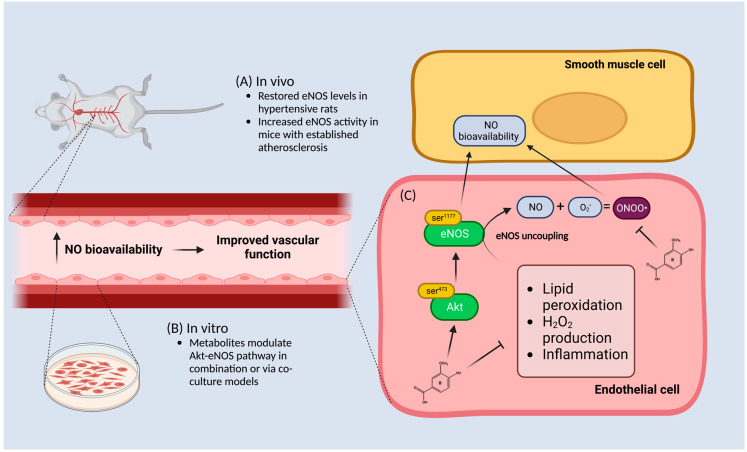
A visual representation illustrating how anthocyanin metabolites improve endothelial function via the Akt-endothelial nitric oxide synthase (eNOS) pathway. (**A**) In vivo data suggests that isolated metabolites restore eNOS levels in hypertensive rats or increase its activity in an established atherosclerosis model. (**B**) In vitro data indicates that isolated metabolites activate the Akt-eNOS pathway in co-culture models or in a mixture with other metabolites in an unstimulated environment. (**C**) Phenolic metabolites in isolation can modulate and induce NO bioavailability in smooth muscle cells via an increase in the akt-pathway which is downstream of eNOS in response to inflammatory stimulators including lipid peroxidation as well as H_2_O_2_ stimulation. Metabolites are also able to modulate eNOS uncoupling by preventing peroxynitrite (ONOO) formation by scavenging reactive oxygen species such as superoxide (O_2_). The result of increased NO bioavailability improves vascular function. Figure was created with Biorender.com.

Briefly, a variety of anthocyanins, usually in their glycoside form, have been shown to increase eNOS via an Akt calcium-independent pathway in ECs, known to improve cell survival and maintain NO bioavailability [43,44]. However, many studies incorporate prolonged incubation periods of cultured cells of >4 h with anthocyanin glycosides at physiological pH, which possibly results in the formation of degradation products [15]. A previous study showed a loss of up to 96% for anthocyanin C3G after a 4 h incubation period in cell culture medium as well as in epithelial cells, which was associated with an increase in the degradation product PCA [15]. This suggests that the phenolic metabolites could be upregulating the Akt-eNOS pathway and NO production (Figure 2). However, one study found that C3G increased eNOS protein levels, whereas its phenolic metabolites VA and PCA) in isolation did not [21]. The study further reported that VA and PCA elicited reductions in superoxide production, which could subsequently decrease scavenging of NO. This might imply that phenolic metabolites could increase NO bioavailability rather than increase its production [21]. There could also be limitations attributed to assessing metabolites in isolation. A mixture of metabolites derived from ellagitannin increased NO bioavailability by increasing eNOS activation in human aortic ECs in basal unstimulated conditions, whereas in isolation they did not [45]. This suggests that metabolites in combination, which would occur in dietary exposure, is potentially required for the activation of the Akt-eNOS pathway.

In co-culture models, metabolites were not able to induce NO release in ECs, though they were able to scavenge ROS molecules [46,47]. Notably, in vitro studies showed that PCA increases eNOS activity in mouse aortic ECs in a co-culture model with macrophage foam cells, but not in aortic ECs alone [48]. However, in human brain vascular ECs, PCA upregulated eNOS expression and NO generation with a major effect seen in the phosphorylation rather than absolute alterations of protein levels [49]. Many metabolites are major benzoic acid derivatives, allowing them to induce the phosphorylation of Akt [50]. Metabolites derived from ginseng berry extract reduced the intracellular accumulation of lipids and overexpression of endothelin-1 by enhancing Akt-eNOS phosphorylation [51]. Sinapic acid has displayed antihypertensive activity by increasing NO production via Akt-eNOS phosphorylation in the presence of H_2_O_2_ [52].

In animal studies, supplementation with VA restored eNOS expression to normal levels and reduced endothelin-1 in hypertensive rats, which consequently lowered blood pressure and improved cardiac and renal function [53]. Protocatechuic acid also promoted endothelial-dependent vasodilation by increasing eNOS activity in ApoE−/− mice with established atherosclerosis, but not in its wild-type C57BL/6J mice free of atherosclerosis [48]. Alternative mechanisms have also been seen, for example, the administration of GA ameliorated angiotensin II-induced development of hypertension and vascular remodeling in mice. Mechanistically, GA reduced the activity and expression of the immunoproteasome catalytic subunits b2i and b5i, which abolished the degradation of eNOS, leading to the production of NO and improvement of the endothelium [54].

The evidence collected to date suggests that metabolites in isolation might restore eNOS activity as well as NO bioavailability, under pathological rather than normal conditions. It is likely that the induction of inflammation, or an established mode of atherosclerosis, is potentially a prerequisite for phenolic metabolites to promote improved vascular function in vivo. Therefore, more evidence is needed to comprehend the Akt-eNOS pathway’s reaction to a mixture of metabolites in the presence of inflammatory conditions since current evidence is limited.

## 6. Anthocyanin Metabolites and the Adhesion of Monocytes

**Figure 3:**
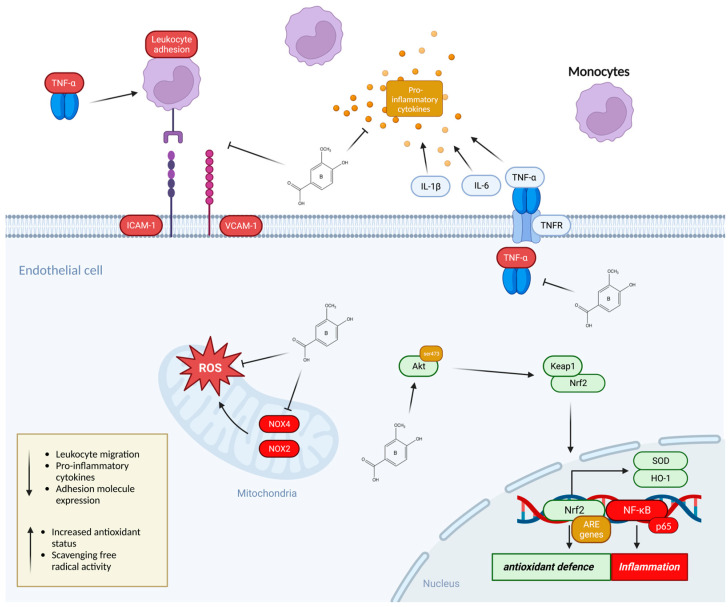
The mechanisms of action by which anthocyanin metabolites prevent the adhesion of monocytes to endothelial cells. Phenolic metabolites can modulate the monocyte adhesion process through activation of Nrf2 transcription factor that increases the antioxidant defence system via the upregulation of antioxidant element (ARE) genes involved in reducing oxidative stress. Phenolic metabolites may also modulate the expression and activity of the NF-κB pathway which is directly linked to the induction of inflammation. NF-κB is known to upregulate the expression of adhesion molecules (vascular cell adhesion molecule (VCAM-1) and intracellular adhesion molecule (ICAM-1)) on the surface of endothelial cells as well as the production of inflammatory cytokines including IL-6, IL-1β, and TNF-α all of which is suppressed by metabolites and contribute to the reduced adhesion of leukocytes including monocytes. Phenolic metabolites also scavenge free radicals by suppressing the expression of NOX2/4 in the mitochondria of endothelial cells.

During the initial stages of atherosclerosis, monocytes accumulate within the vasculature and adhere onto ECs, before differentiating into macrophages. These macrophages remain on the vessel wall, and due to the existence of oxidized lipids, trigger the development of foam cells. However, foam cells cannot leave the vessel wall, leading to chronic inflammation in the local area, promoting the formation of plaques, and raising the likelihood of thrombosis. One type of co-culture model previously used for identifying nutritional components that can mitigate these parameters during the initial stages of atherosclerosis includes the EC–monocyte adhesion assay [55]. In this method, monocyte suspension cells, usually THP-1 or U937 cells, are fluorescently labeled and incubated on top of ECs with anthocyanin or metabolite treatments in the presence of inflammatory cytokines such as TNF-α [55]. The ability of anthocyanins to attenuate monocyte adhesion to ECs has been previously reported in studies, using anthocyanin-rich extracts of glycosides and aglycones but at supra-physiological concentrations (10–200 μM) [56,57,58]. However, more recent studies have shown that the exposure of ECs to individual anthocyanins and their phenolic metabolites, at physiologically relevant concentrations (0.1–2 μM), reduced monocyte adhesion to TNF-α-activated ECs (Figure 3) [59]. Both anthocyanins and gut metabolites have been able to decrease the adhesion of monocytes with a magnitude ranging from 18.1 to 47% compared to TNF-α stimulation [59]. In some cases, where the parent anthocyanin C3G decreased the adhesion by 41.8% at 10 μM, its metabolites reduced the adhesion by 18–59.3% when used at lower concentrations [60].

The mechanisms by which phenolic metabolites reduce the adhesion of monocytes to ECs could be combined with suppressed expression of adhesion molecules (vascular cell adhesion molecule (VCAM-1), intracellular cell adhesion molecule (ICAM-1), or E-selectin), chemokines (MCP-1 or CD40), and a reduction in proinflammatory cytokines (IL-6 or TNF-α) [61,62]. This possibly implicates a prime pharmacological target for controlling inflammatory disease, as the dialogue between ECs and monocytes/macrophages through these key components is a critical event in atherosclerosis [60,61]. Previously, anthocyanin malvidin inhibited TNF-α-induced MCP-1, ICAM-1, VCAM-1, and E-selectin production [62,63]. Moreover, soluble VCAM-1 was suppressed by PCA at 1 μM, and this effect is achievable at relevant concentrations in vivo [64,65]. Reductions in VCAM-1 mRNA have been induced by metabolites only at supra-physiological concentrations (<20 μM), while mixtures of metabolites and flavonoids at physiological concentrations showed no activity [65]. According to the contradictory findings, the method of measuring VCAM-1 or ICAM-1 either via its mRNA expression or in its soluble form may occur as a limitation [65,66]. Membrane-bound VCAM-1 and ICAM-1 do, however, directly bind to leukocytes in the progression of the atherosclerosis process; this is yet to be explored and could be a better marker to verify the association and find out if metabolites interfere with proteolytic cleavage [67,68]. Interestingly, phenolic metabolites seem to not have an active effect on mRNA transcription at physiologically relevant concentrations, and possibly act post-translationally, as suggested by others [65]. Moreover, studies have also used supra-physiological concentrations of TNF-α which may not replicate plasma concentration in vivo and may also explain the contradictory findings [65].

Even though it has been shown that anthocyanins and metabolites at physiologically relevant concentrations decreased the adhesion of TNF-α-stimulated monocytes to ECs, these effects were not mediated by adhesion molecules [59]. Similarly, anthocyanins and metabolites have been found to cause significant reduction of E-selectin, yet not VCAM-1 levels [59]. At lower concentrations, anthocyanins in their original form failed to exert such a protective effect, whereas blueberry metabolites prevented monocyte binding and decreased expression of IL-8, ICAM-1, and VCAM-1 in ECs [69].

Furthermore, metabolites of C3G induced greater reductions in IL-6 and VCAM-1 protein compared to their precursor structure, under both ox-LDL and CD40L stimulation [67,70]. Four combinations of metabolites also reduced TNF-α secretion to a greater extent than the precursors or metabolites alone [71,72]. Given that the PCA reactive catechol moiety is rapidly methylated, it does not persist in systemic circulation, while its metabolite VA exists at much higher concentrations and has a considerably longer half-life. VA may, therefore, be an appropriate target for future investigation since it was recently shown to have an effect in reducing monocyte adhesion through other mechanisms and decreasing VCAM-1 levels [65,72].

## 7. Anthocyanin Metabolites and NF-κB Pathway

Nuclear factor kappa B (NF-κB) is a transcription factor found inactive in the cytoplasm. Its activation is initiated by the signal-induced degradation of IκB proteins, with consequent release of NF-κB into the nucleus where it coordinates the inflammatory process [73]. NF-κB can be found in many cells including ECs, monocytes, and smooth muscle cells [73,74]. Enhanced NF-κB activity is known to cause vascular dysfunction and further develop the progression of atherosclerosis [73]. NF-κB activity mediates the expression of cell adhesion molecules (CAMs), including P-selectin, and VCAM-1 per se leads to an increased interaction between CAMs and antigens on the surface of leukocytes and their adhesion to the vessel wall [75]. Nutrigenomic analysis detected an antiatherogenic effect of anthocyanin metabolites, which is associated with modulation of the expression of genes involved in monocyte adhesion that includes the NF-κB pathway [76]. Caffeic acid at 10 nM, for example, improved intracellular redox status in high-glucose-induced ED by decreasing NF-κB signaling [77,78].

The optimal activation of NF-κB, allowing its binding to the appropriate promoter regions, requires a phosphorylation in the transactivation domain of p65 protein. Thus, in majority of these studies, it was decided to measure the level of p65 phosphorylation as an indication of NF-κB activation. In healthy subjects, after the consumption of daily anthocyanin-rich juice for 28 days, isolated plasma metabolites reduced NF-κB p65 phosphorylation in cancer and ECs in vitro [79].

Protocatechuic acid administration in an ApoE-deficient mouse model reduced aortic VCAM-1 and ICAM-1 by inhibiting atherosclerotic development [80]. Molecular docking revealed that PCA, ferulic acid, and hippuric acid could bind to the domain of p38β [81]. This signaling protein has been linked to p38 mitogen-activated protein kinase (MAPK), which is involved in the regulation of inflammation and cellular responses, and its inhibition is associated with decreased NF-κB activation [81]. Pre-exposure of ECs to a mixture of metabolites significantly reduced NF-κB–p65 phosphorylation and lowered monocyte–EC interaction [58,81]. Flavanol metabolites have also been shown to reduce monocyte adhesion through NF-κB–p65 and p38–MAPK gene expression [81]. Anthocyanin and metabolites led to significant suppression of IL-1β-mediated NF-κB signaling but the MAPK pathway was not involved [82]. VA has previously been reported to suppress the overexpression of NF-κB in lipopolysaccharide-stimulated mouse peritoneal macrophages [83]. However, VA (25, 50, or 100 mg/kg) treatment for 6 weeks significantly downregulated NF-κB expression which exhibited protection against renal impairment in diabetic rats [84]. In humans with metabolic syndrome, 320 mg/day of anthocyanin supplementation for 4 weeks downregulated the expression of NF-κB-dependent genes [85]. To conclude, these studies demonstrate the therapeutic benefits of phenolic metabolites in reducing inflammation and atherosclerosis development and suggests that targeting the NF-κB could be a promising approach for the prevention and treatment of vascular function. Further research is required to determine the optimal dosing and administration.

## 8. Anthocyanin Metabolites and Nrf2 Pathway

The reduction in inflammatory markers is likely mediated by the anthocyanin and metabolite capacity to increase Nrf2 activity [86]. Nrf2 is a transcription factor that is responsible for both the constitutive and inducible expression of the antioxidant response element genes [87]. In a resting state, Nrf2 is sequestered in cytosol and bound with KEAP-1 [86]. More recent theories suggest that anthocyanins could mediate the antioxidant effect through the upregulation of antioxidant genes [87]. It is thought that anthocyanins can protect ECs via direct activation of Nrf2 or through the involvement of extracellular signal regulation kinase [87]. The Nrf2 signaling pathway upregulates antioxidant defenses such as heme oxygenase 1 (HO-1) and superoxide dismutase (SOD) which protect against oxidant injury [87]. Antioxidants or electrophiles such as anthocyanins may directly interact with cysteine residues present in KEAP-1, whereby Nrf2 dissociates and is translocated into the nucleus before binding to ARE or antioxidant enzymes [88]. Vanillic acid, for example, has shown the ability to scavenge free radicals directly, reducing lipid peroxidation, which has been associated with restored SOD activities in the plasma and tissues of diabetic hypertensive rats in response to the negative impact of a high-fat diet [89]. In vitro studies have mainly demonstrated that metabolites increase HO-1 activity through the Nrf2 pathway in ECs [21,89,90]. HO-1 is known to inhibit proinflammatory cytokines IL-6 and TNF-α and the adhesion of monocytes during the initial stages of atherosclerosis [91]. Pretreatment with the phenolic acids derived from blueberries for 18 h resulted in a significant upregulation of the Nrf2-regulated antioxidant response including HO-1 following 6 h exposure to H_2_O_2_ [92,93].

Protocatechuic acid protected ECs against oxidative damage through activating endogenous antioxidant enzymes exploiting an AMPK/Nrf2 pathway [94]. The Nrf2 pathway is also reported to be linked to the inhibition of ROS generation via NADPH in ECs [92]. Anthocyanins can elevate the expression of cytoprotective enzyme HO-1 in ECs with subsequent inhibition of NOX4 activity [89,94,95]. This caused anthocyanins to exhibit high NO levels, which contributed to an effective antioxidant capacity and vasodilatory effect [96]. It is suggested that the glycosylation form of the anthocyanin is the most important factor in determining antioxidant properties of anthocyanin in reducing ROS activity [97]. In most cases, malvidin-3-glucoside had better antioxidant effects than malvidin-3-galactoside and a mixture of anthocyanin elicited similar effects [98]. Generally, glycosylation of an anthocyanin seems to decrease the antioxidant capacity compared with aglycone as it reduces free hydroxyls and metal chelation sites. PCA and its esters revealed high antiradical activity which was probably related to the number of hydroxyl groups present in the PCA molecule [94]. However, metabolites such as PCA being able to activate Nrf2 activity at lower concentrations could explain why it demonstrated a greater effect in reducing IL-6 production; the precursor anthocyanin had no effect on IL-6 production [66,67,98]. VA ameliorated palmitic acid oxidative stress and has been linked to the activation of AMPK/Nrf2 and inhibited mitochondrial dysfunction [90]. Based on these findings, whilst anthocyanins can directly scavenge ROS, phenolic metabolites potentially seem to induce Nrf2 activity and thus could inhibit inflammatory mediators related to ED.

## 9. Implications and Future Directions

As demonstrated throughout the current review, the mechanisms of action by which anthocyanin metabolites modulate endothelial function may differ compared to their original form and cause health promoting effects that can be easily translated to in vivo environments [21]. The limitation with some of the studies explored within the present review is the use of metabolites in isolation, which do not represent a post-prandial situation in vivo; anthocyanin metabolites do not circulate alone but exist as complex mixtures at various concentrations [7,17]. However, different studies cited in the current review demonstrate a synergistic effect when mixing metabolites in contrast to the isolation of individual metabolites. Thus, mixing metabolites is important to consider when designing experiments exploring bioactivity of these phenolic metabolites [99]. Other studies have used both extracted animal and human plasma in a similar manner [100,101,102]; however, the limitation of those studies’ designs is that plasma contains many bioactive constituents other than flavonoid metabolites, making it difficult to compare treatments to controls and eliminate confounders stemming from varying endogenous analytes [101,102].

Furthermore, co-culture models described in the current review could serve as a useful tool for studying the mechanisms of action of anthocyanin metabolites in an atherosclerosis model. Some cells cannot be easily monocultured in vitro or at least do not exhibit in vivo physiological behaviors; however, the availability of another cell population may improve the culturing success and/or cell behavior [103]. Co-culture models can also better stimulate the complex microenvironment in vivo and allow for the examination of the effects of anthocyanin metabolites on multiple cell types simultaneously, providing a more accurate representation of physiological responses. 

In recent research, genetic manipulation, aiming at upregulating the gene expression of metabolic pathways, has been used to improve anthocyanin content in plant foods and potentially would also contribute towards counteracting reduced bioavailability in vivo [104,105,106]. A prominent case in point is the recent study by Riva and co-workers (2019), who used a new delivery system based on food grade lecithin to increase plasma concentration of quercetin by up to 20 times more than usual [104]. Similarly, anthocyanins contained in nanoliposomes have displayed a sustained release and high stability during digestion in vitro [105]. Microencapsulation can protect anthocyanins from degradation and release them slowly in the gastrointestinal tract, improving their bioavailability. This could enhance their potential health benefits and have important implications for CVD prevention and treatment.

## 10. Conclusions

Anthocyanins have been shown to be effective at restoring endothelial function during the initial stages of atherosclerosis and, more specifically, ED. Recent and progressive data using physiological concentrations of phenolic metabolites in vitro have helped to gain more specific knowledge and further understanding of the mechanisms of action within the vasculature. These findings have facilitated better understanding of the use of anthocyanins in the treatments of vascular diseases, including atherosclerosis. Overall, it is concluded that metabolites of anthocyanins can improve vascular health at concentrations that can be achieved in vivo. More research investigating anthocyanin metabolites in synergistic actions is recommended with key considerations to improve their bioavailability and usage as a nutraceutical supplement.

## Data Availability

No new data were created or analyzed in this study. Data sharing is not applicable to this article.

## Abbreviations

Augmentation index (Aix), cell adhesion molecules (CAMs), cyanidin-3-glucoside (C3G), cardiovascular disease (CVD), endothelial cells (EC), endothelial dysfunction (ED), endothelial nitric oxide synthase (eNOS), flow-mediated dilation (FMD), gallic acid (GA), heme oxygenase 1 (HO-1), intracellular cell adhesion molecule (ICAM-1), mitogen-activated protein kinase (MAPK), nuclear factor kappa B (NF-κB), nitric oxide (NO), protocatechuic acid (PCA), pulse wave velocity (PWV), reactive oxygen species (ROS), superoxide dismutase (SOD), vanillic acid (VA), and vascular cell adhesion molecule (VCAM-1).
